# Liver resection for HER2-enriched breast cancer metastasis: case report and review of the literature

**DOI:** 10.1186/s40792-017-0307-1

**Published:** 2017-02-21

**Authors:** Mai Temukai, Hajime Hikino, Yoshinari Makino, Yoko Murata

**Affiliations:** 1 Department of Breast Surgery, Matsue Red Cross Hospital, 200 Horo, Matsue, Shimane 690-8506 Japan; 2 Department of Breast and Endocrine Surgery, Tottori University, 36-1, Nishimachi, Yonago, Tottori 683-8504 Japan

**Keywords:** Breast cancer, Liver metastasis, Hepatectomy

## Abstract

Liver metastasis from breast cancer usually results in the development of systemic metastasis. We report a breast cancer patient with an early isolated liver recurrence who survived more than 7 years with no recurrence. She was treated with aggressive HER2-directed chemotherapy and hepatic metastasectomy. Local hepatectomy with effective medical oncological therapy with curative intent is worth trying in patients with breast cancer liver metastasis.

## Background

The liver is the primary site of recurrence in 12–15% of breast cancer patients [1]. Under routine imaging workup such as positive emission tomography (PET) and computed tomography (CT), the incidence of an isolated liver metastasis has been reported as approximately 0.9% among patients who underwent breast cancer surgery [2]. Even in patients with an isolated liver metastasis at the first presentation, breast cancer liver metastasis (BCLM) is usually part of generalized metastatic disease, resulting in the development of systemic metastasis. If treated with chemotherapy, the median survival of breast cancer patients with only liver metastasis or with limited disease elsewhere is 19 to 26 months [3]. For the select subset of patients presenting with isolated BCLM, hepatic metastasectomy has been proposed as a useful treatment associated with a good outcome [4]. However, the strategy of hepatic metastasectomy in BCLM is still debatable. We present a patient who developed a solitary BCLM as the first recurrence and yet achieved long-term disease-free survival after aggressive human epidermal growth factor receptor 2 (HER2)-directed chemotherapy and hepatic metastasectomy.

## Case presentation

A 56-year-old woman was referred to our hospital with a 1-month history of a lump in her left breast. Physical examination revealed a 4.0 × 3.0-cm tumor in the lower outer quadrant of the left breast. Biopsy specimen by core needle biopsy from the left breast lump showed an invasive ductal carcinoma that was both estrogen receptor (ER)- and progesterone receptor (PgR)-negative with a HER2 of 3+ by immunohistochemistry assay. A PET/CT scan showed uptake of 18F-fluorodeoxyglucose only in the left breast tumor and the left axillary lymph nodes, but not in distant organs. The patient was diagnosed with primary breast cancer (cT2N1M0 stage IIB according to the Union for International Cancer Control (UICC) classification).

She received preoperative chemotherapy consisting of paclitaxel (80 mg/m^2^) weekly for 12 weeks, followed by 5-fluorouracil, epirubicin, and cyclophosphamide (FEC) (500/100/500 mg/m^2^) four times every 3 weeks. Following preoperative chemotherapy, physical examination revealed only induration of the left breast. Preoperative chest and abdominal CT scanning with contrast medium showed no signs of distant metastasis.

The patient underwent a modified radical mastectomy of the left breast. Pathological evaluation confirmed residual invasive ductal carcinoma within the breast tissue (ER- and PgR-negative, HER2 3+) and metastatic carcinoma within three dissected axillary lymph nodes.

**Fig. 1:**
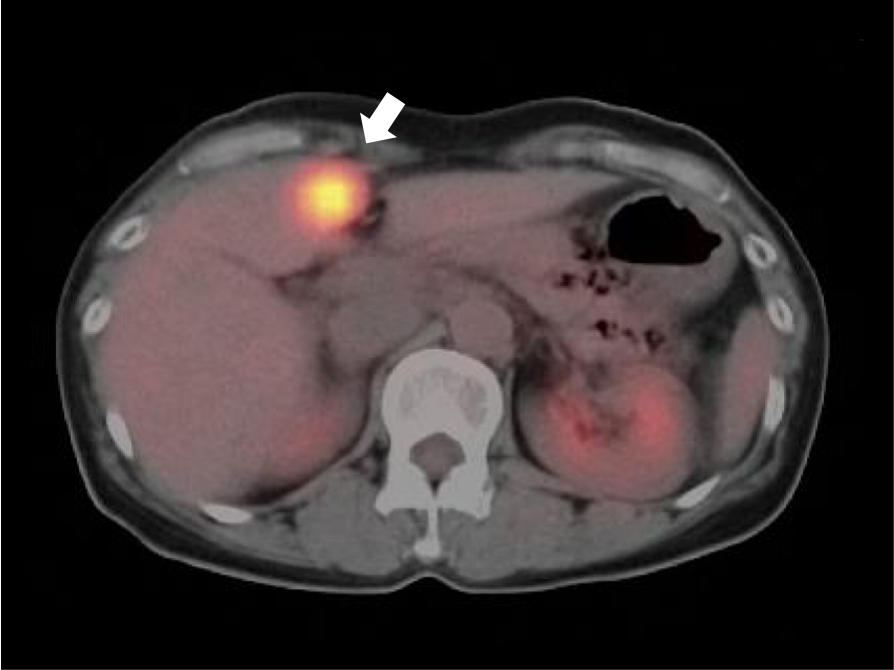
Abdominal computed tomography. Postoperative abdominal computed tomography revealed an ill-defined mass (*white arrow*), 16 × 18 mm in diameter, on liver segment IV

Following surgery, the patient received weekly trastuzumab monotherapy (initially 4 mg/kg, followed by two or more cycles of 2 mg/kg) as adjuvant treatment. Three months after the start of trastuzumab, the patient’s serum HER2 level had increased to 17.6 ng/ml (normal range <15.2 ng/ml). Metastatic workup with PET/CT scan and sonography of the liver revealed an isolated liver metastasis (segment IV, 16 × 18 mm in diameter) (Fig. 1). Weekly trastuzumab and vinorelbine ditartrate (25 mg/m^2^, day 1, 8, every 3 weeks) were administered as the first-line treatment, but the tumor did not respond. Then, weekly trastuzumab and triweekly docetaxel (60 mg/m^2^) were administered as the second-line treatment. However, 2 months later, the liver metastasis had progressed. We modified the chemotherapeutic regimen to oral capecitabine (1650 mg/m^2^, day 1–14, every 21 days) along with trastuzumab and triweekly docetaxel as the third-line treatment. After 4 cycles of this chemotherapy, CT scanning showed that the size of the liver metastasis had decreased. However, soon after, progression of the remaining liver metastasis was anticipated from her clinical course.

**Fig. 2:**
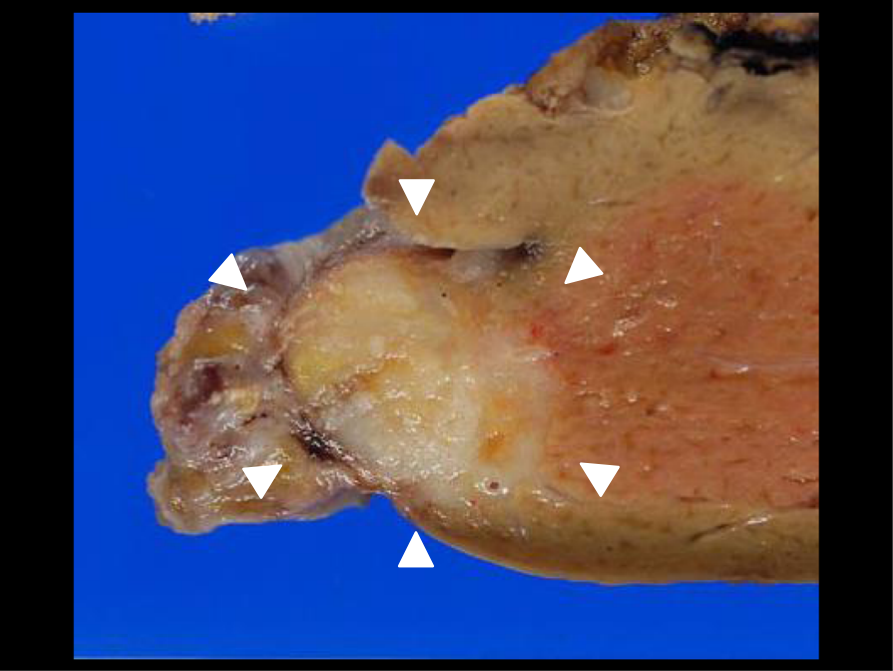
Gross view of liver tumor. Gross view of liver tumor (*white arrowheads*) shows a yellowish-white, elastic-hard solid component, 7 × 9 mm in diameter

After adequate informed consent, the patient underwent partial resection of the liver (R0) (Fig. 2). The tumor was consistent with liver metastasis (7 × 9 mm) from breast cancer and showed the same pathological characteristics: ER- and PgR-negative, HER2 3+. After liver metastasectomy, the patient was administered a combined chemotherapy regimen of weekly trastuzumab and oral capecitabine. Two months later, her cardiac function had deteriorated as an adverse effect of the chemotherapy. Although she has not been treated thereafter, no local recurrence or other distant metastases have appeared for more than 7 years.

### Discussion

The patient described here had favorable long-term survival after diagnosis of liver metastasis, which may demonstrate that a select group of patients with isolated liver metastasis can benefit from aggressive systemic HER2-directed chemotherapy and complete surgical resection, even without continuous chemotherapy.

Weichselbaum and Hellman proposed that a state of oligometastasis may exist in which the metastases are limited in number and site [5]. Good outcome has been observed in patients treated with local therapy for oligometastasis. Although local hepatectomy is possible for solitary BCLM, many patients develop recurrent disease [4]. In a study by Lermite et al., liver recurrences were diagnosed at a mean interval of 15 months post-hepatectomy and extrahepatic recurrences at 22 months [6]. Ruiterkamp and Ernst found that in 13–56% of all cases in their study, the first location of recurrence was in the remaining liver [7]. It is not possible to predict the subsequent clinical course of disease, or to determine whether the presence of limited liver metastasis represents a true state of oligometastasis or a transitional state to disseminated metastasis. In the present case, early hepatic recurrence developed at 3 months after mastectomy—during adjuvant trastuzumab therapy—fitting into the latter scenario. Achievement of only local control of liver metastasis could rarely be “curative.” Co-administration of effective medical oncological therapy may be needed for long-term survival.

In the literature, several independent factors have been proposed that might influence the prognosis of breast cancer patients after liver metastasectomy (Table 1). A literature search was performed through the Medical Literature Analysis and Retrieval System Online (MEDLINE) database. Fisher et al. first reported in 1990 the efficacy of the Adriamycin and cyclophosphamide (AC) regimen, which today is one of the main prescribed chemotherapy regimens for breast cancer [8]. Therefore, we selected reports that were published after 1990. As a general problem, most published studies are designed as retrospective single-arm and single-center analyses, analyzing data of only small sample sizes that were collected over periods of ≥10 years. Our literature search showed that there is no clear consensus on selection criteria for referral of patients for liver metastasectomy.

**Table 1:** Prognostic factors of overall survival in patients after liver metastasectomy from breast cancer. (only multivariate Cox regression analyses)

Study	Hoffman^10^	Abott^15^	Walsum^12^	Zegarac^13^	Dittmar^16^	Weinrich^9^	Vertriest^14^
Number	41	86	32	32	34	21	27
Duration	1999–2008	1997–2010	1991–2011	2006–2009	1997–2010	2001–2007	1996–2013
Median overall survival (months)	58	57	55	37	36	53	116
5-year overall survival (%)	48.4		37		28	33	78
Median disease-free survival (months)	34	14.2	11	22.5			
5-year disease-free survival (%)	31		19				36
Characteristics of primary BC							
ER status		*p* = 0.009		*p* < 0.05			
		(pos)		(pos)			
Lymph node status				*p* < 0.05			
				(neg)			
Tumor size				*p* < 0.05			
				(<3 cm)			
Grading						*p* = 0.0059	
Stage							*p* = 0.03
							(Stages I–II)
Characteristics of LM and LM treatment							
Age					*p* = 0.004		
					(<50)		
Period from BC to LM	*p* = 0.0097			*p* < 0.05			
	(>24 months)			(>24 months)			
Number of LM			*p* < 0.05	*p* < 0.01			*p* = 0.04
			(1 vs >1)	(1 vs >1)			(1 vs >1)
HER2 status					*p* = 0.010		
					(pos)		
Absence of extrahepatic tumor					*p* = 0.042		
Response to preoperative chemotherapy		*p* = 0.003					
		(good response)					
Resection margins	*p* = 0.0015						
	(R0 vs R1/2)						

*BC* breast cancer, *ER* estrogen receptor, *LM* liver metastasis, *HER2* human epidermal growth factor receptor 2, *neg* negative, *pos* positive, *R0* complete microscopic resection, *R1* microscopic residual disease, *R2* macroscopic residual hepatic or extrahepatic disease

With this background, most authors have described achievement of R0 (complete macroscopic and microscopic) resection as the main prognostic factor for breast cancer patients after liver metastasectomy [9, 10]. Two selection criteria for patients to be accepted for curative hepatic resection are, first, the absence of extrahepatic disease (with the exception of isolated pulmonary and bony metastases) and, second, the ability of the surgeon to perform an R0 resection with a low risk of morbidity [11]. Furthermore, solitary liver metastasis is a significant prognostic factor [12–14]. Preoperative detailed exploration using multislice CT, contrast-enhanced MRI, or PET scan should be mandatory to minimize unnecessary surgery and maximize survival benefit.

The second prognostic factor for breast cancer patients after liver metastasectomy is a long interval (more than 1 year) between diagnosis of breast cancer and detection of liver metastasis [6, 7, 10, 13]. Several studies have found that patients with ER- and/or PgR-positive BCLM are good candidates for liver metastasectomy due to favorable tumor biology and administration of hormone therapy [13, 15]. Such characteristics of primary breast cancer as small tumor size, node negativity, low grade and early stage may also be associated with better outcome after liver metastasectomy [9, 13, 14]. On the other hand, patients with triple negative phenotype in the primary breast cancer and/or liver metastases may not benefit from liver metastasectomy due to aggressive biological behavior and limited treatment strategies.

The third factor proposed as influencing the prognosis of breast cancer patients after metastasectomy is response to preoperative systemic therapy [15]. If systemic therapy can eradicate microscopic metastatic tumor in remnant liver and systemic organs, aggressive local therapies to treat limited BCLM may translate into improved survival. The response may also serve as a guide for further postoperative therapy. On the other hand, the patient who has not demonstrated disease regression or stability with systemic chemotherapy may not be a candidate for liver metastasectomy. Overexpression of HER2 on hepatic metastases may be correlated with improved survival [16, 17]. Pooled discordance proportion between primary and recurrent breast cancer was reported in 8% of patients with HER2, 20% of patients with ER, and 33% of patients with PgR by a meta-analysis [18]. Furthermore, it is suggested that the mutation of the metastatic phenotype is lower at earlier recurrence sites. Actually, no biological change was demonstrated between primary and corresponding asynchronous liver metastasis in the present patient. Response-guided chemotherapy for liver metastasis may also be effective in systemic micro-metastasis, contributing to long-term survival with no continuous chemotherapy. Because of remarkable progress in the effectiveness of HER2-directed therapy, the use of local therapy to treat limited BCLM may expand further in patients with overexpression of HER2 on hepatic metastases [6].

## Conclusions

Liver metastasectomy may offer a survival advantage over systemic chemotherapy alone in select patients with BCLM. However, independent prognostic factors predictive for survival are still not clearly defined. Further studies are needed to identify a subgroup of patients who may benefit from aggressive multidisciplinary treatment including liver metastasectomy.

## Abbreviations

BCLM: Breast cancer liver metastasis
ER: Estrogen receptor
HER2: Human epidermal growth factor receptor 2
PgR: Progesterone receptor
